# Admission inflammatory and immuno-nutritional indices and in-hospital mortality in emergency department patients admitted to intensive care: a retrospective cohort study

**DOI:** 10.3389/fmed.2026.1903440

**Published:** 2026-07-20

**Authors:** Mustafa Enes Demirel, Ahmet Celal Çapkan, Samet Sezer Can, Muhammed Ali Binzet

**Affiliations:** 1 Department of Emergency Medicine, Medical Faculty, Bolu Abant İzzet Baysal University, Bolu, Türkiye; 2 Department of Interdisciplinary Biotechnology, Institute of Graduate Education, Bolu Abant İzzet Baysal University, Bolu, Türkiye; 3 Department of Family Medicine, Medical Faculty, Bolu Abant İzzet Baysal University, Bolu, Türkiye; 4 Medical Faculty, Bolu Abant İzzet Baysal University, Bolu, Türkiye

**Keywords:** C-reactive protein-to-albumin ratio, critical illness, emergency department, inflammation, multi-inflammatory index, intensive care unit, mortality

## Abstract

**Background:**

This study investigated whether admission-based composite inflammatory indices and C-reactive protein-to-albumin ratio (CAR) were associated with in-hospital mortality in adult patients admitted from the emergency department to the intensive care unit.

**Methods:**

This retrospective cohort study included 709 adult patients admitted to the intensive care unit (ICU) via the emergency department. Inflammatory and immuno-nutritional indices at the time of admission, including C-reactive protein/albumin ratio (CAR) and three Multi-Inflammatory Index variants were evaluated. MII-1, MII-2, and MII-3 were calculated as neutrophil to lymphocyte ratio × CRP, platelet to lymphocyte ratio × CRP, and systemic immune-inflammation index × CRP, respectively. Associations with in-hospital mortality were assessed using unadjusted analyses, age- and sex-adjusted Cox regression models and descriptive receiver operating characteristic analyses.

**Results:**

Non-survivors exhibited significantly higher median MII values and CAR levels compared with survivors in unadjusted analyses (*p* < 0.001). In SD-scaled age- and sex-adjusted Cox analyses, CAR and all three MII variants were associated with in-hospital mortality. However, ROC analyses showed limited discriminatory performance for these indices. CAR showed the most consistent association with mortality, including in the sensitivity analysis adjusted for GCS score and oncologic disease status. Discriminative performance was modest for all indices, with CAR showing higher area under the curve values (AUC: 0.627) compared with MII variants (AUC range: 0.579–0.595).

**Conclusion:**

MII variants and CAR were associated with mortality in admission-based analyses. However, their discriminatory performance was limited. Overall, these findings support the interpretation of admission-based inflammatory indices as adjunctive markers of inflammatory burden rather than standalone prognostic tools in emergency-to-ICU patients.

## Introduction

Emergency departments (EDs) are an essential component of the healthcare system where time-sensitive diseases with high mortality risk are first encountered and managed (1). In this dynamic environment, early and accurate risk stratification is particularly important for identifying critically ill patients who may require intensive care unit (ICU) admission (2). Patients referred to the ICU frequently present with systemic inflammation and organ dysfunction (3). Therefore, the early identification of patients at increased risk of adverse outcomes, such as in-hospital mortality and prolonged hospitalization, remains a clinically relevant challenge in emergency medicine. In this context, there is a recognized need for rapid, inexpensive, and easily accessible prognostic markers that may support early clinical decision-making in the emergency department.

The accuracy of assessments performed at initial presentation in the ED is often associated with important clinical outcomes such as mortality, morbidity and length of hospitalization. In this context, the prognostic value of laboratory parameters used for accurate triage of critically ill patients has been increasingly investigated (4, 5). The need for rapid decision-making in the ED leads clinicians to use biomarkers that are both rapidly accessible and have potential clinical utility. Accordingly, neutrophil count, platelet level and C-reactive protein (CRP), which are indicators of systemic inflammation, are among the most frequently used laboratory parameters (4, 6). These parameters may reflect the intensity of the acute inflammatory response and have been associated with outcomes in selected clinical conditions (6–8).

Systemic inflammation is common in critically ill patients admitted from the emergency department to the intensive care unit. While routinely available markers such as neutrophil count, platelet count, CRP, and albumin provide useful information, they may not fully reflect the complex inflammatory and nutritional status of critically ill patients. Therefore, composite indices integrating hematological, inflammatory, and nutritional parameters are increasingly being investigated as exploratory tools for early risk stratification (3, 9, 10).

Critical illness is a complex process involving multiple etiologies and risk factors, often following acute and chronic diseases. Systemic inflammatory responses play an important role in this process. In this response, CRP, the main acute phase protein, and albumin (ALB), the most abundant protein in plasma, are markers commonly used in the clinic to assess inflammation and nutritional status (11, 12). Especially albumin is a widely used indicator of nutritional reserve and general health status (12–14). Furthermore, the C-reactive protein/albumin ratio (CAR) is a recently proposed inflammatory prognostic marker associated with outcomes in critical illnesses, particularly sepsis and organ dysfunction, and in selected oncological populations (13, 15, 16).

The Multi-Inflammatory Index (MII) is a composite inflammatory index derived from neutrophil, lymphocyte, platelet, and CRP values. In this study, three MII formulations were evaluated: MII-1, calculated as neutrophil to lymphocyte ratio × CRP; MII-2, calculated as platelet to lymphocyte ratio × CRP; and MII-3, calculated as systemic immune-inflammation index × CRP (17). Each of these formulations represents a different combination of CRP with different hematological dimensions of the inflammatory response. These formulations were evaluated not as competing predictive models, but as related exploratory markers reflecting overlapping aspects of systemic inflammation. Accordingly, differences in performance between MII-1, MII-2, and MII-3 should not be interpreted as evidence of superiority, but as reflecting overlapping biological processes captured through distinct mathematical constructions (17–20). In particular, whether MII offers added value over routinely used indices such as the CAR has not been clearly established in critically ill populations. Therefore, the present study was designed to explore the association of admission-based MII and CAR values with in-hospital mortality and length of stay among adult patients admitted to the ICU from the emergency department, with an explicitly exploratory and hypothesis-generating framework rather than as a validation of a novel prognostic tool.

## Materials and methods

### Study population

This study was conducted as a retrospective single-center cohort study. Data were obtained from the medical records of adult patients who presented to the emergency department of a tertiary care university hospital between January 1, 2021, and December 31, 2024, and were subsequently admitted to the intensive care unit. Ethical approval for this retrospective study was obtained from the Local Clinical Research Ethics Committee [Decision date: 12.08.2025, No: 2025/319]. The study was conducted in accordance with the principles of the Declaration of Helsinki. Data were obtained only from retrospective patient records and no intervention was made. Patient names and identity information were kept confidential and anonymized data were used.

### Study design and data collection

Adult patients aged 18 years and older who were first evaluated in the ED and then hospitalized in the ICU were included in the study. Demographic data, admission diagnoses recorded in the hospital information system, laboratory parameters obtained at ED presentation, intensive care unit hospitalization details, and clinical outcomes were retrospectively extracted from the electronic hospital records and ICU follow-up files. The study was reported in accordance with the STROBE guidelines. The requirement for informed consent was waived due to the retrospective design.

Since this study was conducted in a general intensive care unit that admitted patients from the emergency department, patients with primary cardiac or neurological indications were generally referred to the relevant specialized intensive care units. For descriptive purposes, length of hospital stay, ICU stay, and major ICU interventions were summarized. Primary admission categories included respiratory failure, sepsis or infection-related conditions, renal-metabolic disorders, and decreased level of consciousness without a primary neurological diagnosis. Laboratory parameters were obtained from initial blood samples taken upon emergency department admission prior to transfer to the intensive care unit. When multiple measurements were available, the earliest recorded value was used. Complete blood counts and biochemical parameters were performed in the hospital’s central laboratory using standard automated analyzers in accordance with routine institutional protocols. The inflammatory and immuno-nutritional indices evaluated in this study were calculated using previously validated formulas and are summarized in Table 1.

**Table 1:** Definitions and formulas of inflammatory and immuno-nutritional indices used in the study.

Index	Full name	Formula
NLR	Neutrophil-to-Lymphocyte Ratio	Neutrophil count (10^3^/μL) ÷ Lymphocyte count (10^3^/μL)
PLR	Platelet-to-Lymphocyte Ratio	Platelet count (10^3^/μL) ÷ Lymphocyte count (10^3^/μL)
SII	Systemic Immune-Inflammation Index	[Neutrophil count (10^3^/μL) × Platelet count (10^3^/μL)] ÷ Lymphocyte count (10^3^/μL)
SIRI	Systemic Inflammation Response Index	[Neutrophil count (10^3^/μL) × Monocyte count (10^3^/μL)] ÷ Lymphocyte count (10^3^/μL)
AISI	Aggregate Index of Systemic Inflammation	[Neutrophil count (10^3^/μL) × Platelet count (10^3^/μL) × Monocyte count (10^3^/μL)] ÷ Lymphocyte count (10^3^/μL)
MII-1	Multi-Inflammatory Index-1	Neutrophil count (10^3^/μL) ÷ Lymphocyte count (10^3^/μL) × CRP
MII-2	Multi-Inflammatory Index-2	Platelet count (10^3^/μL) ÷ Lymphocyte count (10^3^/μL) × CRP
MII-3	Multi-Inflammatory Index-3	[Neutrophil count (10^3^/μL) × Platelet count (10^3^/μL)] ÷ Lymphocyte count (10^3^/μL) × CRP
CAR	C-reactive Protein-to-Albumin Ratio	CAR = CRP (mg/L) ÷ Albumin (g/L)
HALP	Hemoglobin-Albumin-Lymphocyte-Platelet Score	[Hemoglobin (g/L) × Albumin (g/L) × Lymphocyte count (10^9^/L)] ÷ Platelet count (10^9^/L)
PNI	Prognostic Nutritional Index	[10 × Albumin (g/dL)] + [0.005 × Lymphocyte count (/mm^3^)]
PMI	Platelet Mass Index	Platelet count (10^3^/μL) × Mean Platelet Volume (fL)

Laboratory values were converted to the units specified in the formulas where required.

APACHE II and SOFA scores could not be reliably calculated because some physiological variables necessary for accurate scoring in the first 24 h of ICU admission were not consistently available in retrospective records. Simpler admission-based severity scores were also evaluated. However, baseline variables such as respiratory rate and other physiological measurements were not consistently available across the entire cohort. Therefore, CAR or MII variants could not be compared with validated severity scores in the present analysis. Patients with hematological malignancies were excluded because leukemia, lymphoma, and related conditions directly affect absolute neutrophil, lymphocyte, and platelet counts, essential components of all MII formulations making composite index calculations unreliable. Patients with solid tumors were included in the study if they were not receiving active myelosuppressive chemotherapy at admission, as a solid tumor diagnosis alone is not expected to systematically affect CBC-based inflammatory indices. However, the higher prevalence of oncological disease observed in non-survivors may reflect, in part, the prognostic burden of malignancy rather than the inflammatory index values, and this should be considered when interpreting the results.

### Admission-based assessment rationale

The present study was specifically designed to evaluate inflammation-based indices derived from laboratory parameters obtained at the time of emergency department admission. This approach reflects real-world clinical practice, where early risk stratification and triage decisions must often be made before longitudinal data become available. Therefore, the analysis focused on admission-based measurements rather than serial biomarker trajectories, with the aim of assessing the prognostic relevance of readily accessible indices during the earliest phase of critical illness.

All three MII variants were treated as equally valuable exploratory indices; no MII formulation was predetermined as the primary MII endpoint, and performance differences between variants were not interpreted as evidence of superiority.

### Inclusion criteria

ICU hospitalization after admission to the emergency department.

Hospitalization process completed and clinical outcome (discharge or death) recorded.

### Exclusion criteria

Patients admitted by transfer from another institution and whose initial laboratory data were obtained at an external center,

Being hospitalized in the ICU of another hospital.

Patients with hematological malignancy (leukemia, lymphoma, etc.) or receiving immunosuppressive chemotherapy.

Patients with multiple organ injuries due to trauma.

Patients younger than 18 years of age.

### Statistical analysis

The distribution of continuous variables was assessed using the Shapiro–Wilk test (21). Variables with a normal distribution were expressed as mean ± standard deviation, whereas non-normally distributed variables were presented as median (interquartile range, IQR). As most continuous variables did not meet the assumptions of normal distribution, comparisons between two groups were performed using the Mann–Whitney U test, with corresponding U statistics reported, while categorical variables were compared using the chi-square (χ^2^) test and expressed as number and percentage [n (%)]. The primary clinical endpoint of the study was in-hospital mortality. Length of stay was considered a secondary descriptive endpoint and was not considered a primary prognostic endpoint. CAR and the three MII variants were considered as the primary inflammatory indices of the study; other hematological, inflammatory, and immuno-nutritional indices, including NLR, PLR, SII, SIRI, AISI, HALP, PNI, and PMI, were considered as exploratory comparison indices. The primary clinical endpoint of the study was in-hospital mortality; discharge was considered a competing event. All three MII variants (MII-1, MII-2, and MII-3) were evaluated as equally important exploratory indices; no MII formulation was predetermined as the primary MII endpoint, and performance differences between variants were not interpreted as evidence of superiority. CAR was considered the primary index of interest based on previous literature demonstrating its association with mortality in critically ill patient populations. Overall, the analyses were exploratory and hypothesis-forming, not confirmatory. Inferential analyses primarily focused on in-hospital mortality. Length of stay was analyzed descriptively as a secondary outcome and was not used as a primary prognostic endpoint.

Cause-specific Cox proportional hazards regression models were used to evaluate the association between inflammatory indices and in-hospital mortality, accounting for the competing risk of discharge. Primary multivariable analyses were adjusted for age and sex. Extensive clinical adjustment was not used in the main models because detailed admission diagnostic categories and validated disease severity scores were not consistently available in standardized retrospective medical records. Results were reported as cause-specific hazard ratios (csHRs) with 95% confidence intervals (CIs). To improve interpretability for indices with large numerical ranges, additional SD-scaled cause-specific Cox analyses were performed. In these analyses, hazard ratios for inflammatory and immuno-nutritional indices were reported per 1 standard deviation increase. Prior to SD scaling, all indices were winsorized at the 99th percentile to reduce the potential influence of extreme outliers on variance estimates and model stability. Variables occurring during the ICU stay, including dialysis requirement, sepsis, inotropic support, mechanical ventilation, and acute kidney injury, were not included as predictors in any prognostic Cox regression model to avoid temporal bias and information leakage. As a sensitivity analysis, an extended cause-specific Cox model was constructed by adding GCS score and oncologic disease status to the primary age- and sex-adjusted model. Albumin was not included in this model to avoid collinearity, because albumin is a direct component of the CAR formula.

The pre-defined primary analysis assessed the relationship between inflammatory indices at admission and in-hospital mortality. ROC analyses, cutoff value calculations based on the Youden index, length-of-stay comparisons, and additional subgroup or descriptive comparisons were considered as exploratory post-hoc analyses. The discriminative performances of inflammatory and nutritional indices for in-hospital mortality and length of stay were evaluated using receiver operating characteristic (ROC) curve analysis, with area under the curve (AUC) values calculated. AUC values were interpreted as follows: 0.90–1.00, excellent; 0.80–0.89, good; 0.70–0.79, fair; 0.60–0.69, poor; and <0.60, unacceptable. Optimal cut-off values were determined using the Youden index, and corresponding sensitivity and specificity values were calculated. In all analyses, statistical significance was assessed using two-tailed *p* values, with effect estimates reported together with their corresponding 95% confidence intervals. All statistical analyses were performed using SPSS software (version 21; IBM Corp., Armonk, NY, USA).

## Results

A total of 709 patients admitted from the emergency department to the ICU were included in the analysis. Of these, 478 patients (67.4%) survived and 231 patients (32.6%) died during hospitalization.

### Cohort characteristics

Baseline demographic characteristics, comorbidities, laboratory parameters, inflammatory indices, and intensive care unit-related variables stratified by survival status are presented in Table 2.

**Table 2:** Comparison of baseline characteristics between survival and death groups.

Variables	Survivor (*n* = 478)	Death (*n* = 231)	*p*-value	Test	Statistic
Age	69.77 ± 16.89	75.34 ± 13.25	**<0.001**	U	45,167
Gender n (%) Female	220 (66.1%)	113 (33.9%)	0.52	Χ^2^	0.4134
Gender n (%) Male	258 (68.6%)	118 (31.4%)			
LOS (days)	4.0 [2.0–10.0]	7.0 [2.0–17.0]	0.131	U	51,364
DM n (%)	176 (36.8%)	96 (41.6%)	0.257	Χ^2^	1.2851
HT n (%)	340 (71.1%)	179 (77.5%)	0.089	Χ^2^	2.8948
CAD n (%)	199 (41.6%)	122 (52.8%)	0.007	Χ^2^	7.4146
COPD n (%)	99 (20.7%)	45 (19.5%)	0.778	Χ^2^	0.0796
HF n (%)	74 (15.5%)	45 (19.5%)	0.219	Χ^2^	1.5086
OnkoDis n (%)	35 (7.3%)	32 (13.9%)	**0.008**	Χ^2^	7.0178
GCS	14.0 [8.0–15.0]	8.0 [3.0–13.0]	**<0.001**	U	77536.5
Urea (mg/dL)	60.5 [37.0–100.75]	88.0 [54.0–139.0]	**<0.001**	U	39,873
Crea (mg/dL)	1.09 [0.79–1.74]	1.64 [0.92–2.77]	**<0.001**	U	41,318
eGFR (mL/min/1.73 m^2^)	60.95 [31.32–88.47]	34.46 [18.64–66.76]	**<0.001**	U	70996.5
AST (U/L)	28.0 [18.0–50.0]	46.5 [26.0–159.75]	**<0.001**	U	37335.5
ALT (U/L)	20.0 [12.0–39.5]	28.0 [14.0–75.0]	**<0.001**	U	45805.5
WBC (×10^3^/μL)	11.36 [8.25–16.4]	12.14 [8.18–16.9]	0.391	U	53,017
HGB (g/dL) Mean ± SD	11.3 ± 2.5	10.62 ± 2.56	**0.001**	U	63,534
PLT (×10^3^/μL)	209.5 [159.25–278.5]	185.0 [124.0–251.5]	**<0.001**	U	64209.5
RDW (fL)	14.6 [13.5–16.48]	16.25 [14.6–18.6]	**<0.001**	U	38033.5
LYM (×10^3^/μL)	0.95 [0.63–1.5]	0.85 [0.51–1.3]	**0.006**	U	62193.5
MONO (×10^3^/μL)	0.66 [0.39–0.98]	0.58 [0.28–0.92]	0.068	U	59878.5
NEU (×10^3^/μL)	9.41 [6.45–13.8]	10.13 [6.44–14.65]	0.211	U	52,012
PDW (%)	12.3 [10.7–14.12]	12.7 [11.2–14.9]	0.015	U	44,918
MPV (fL)	10.7 [10.0–11.5]	10.9 [10.3–11.8]	**0.009**	U	44473.5
PCT (%)	0.23 [0.18–0.29]	0.21 [0.15–0.28]	**0.016**	U	56593.5
CRP (mg/L)	41.85 [10.35–114.08]	83.5 [27.55–168.3]	**<0.001**	U	43487.5
Albumin (g/L)	33.0 [28.0–38.0]	29.0 [23.0–33.0]	**<0.001**	U	74701.5
NLR	10.04 [5.43–17.82]	11.46 [6.44–22.26]	**0.012**	U	48787.5
PLR	216.94 [123.7–355.7]	221.57 [123.0–388.4]	0.820	U	54,627
HALP	17.19 [9.44–30.42]	13.81 [6.785–27.57]	0.004	U	62,646
SII	2046.46 [950.4–4064.82]	2055.24 [1031.29–4703.14]	0.650	U	54,048
AISI	1127.21 [488.3–2970.27]	1133.43 [329.72–3243.5]	0.520	U	56,856
SIRI	5.45 [2.68–12.76]	6.16 [2.34–14.64]	0.472	U	53,372
MII-1	433.9 [63.21–2016.16]	1145.6 [125.26–4814.88]	**<0.001**	U	43,838
MII-2	8951.64 [1597.38–39139.65]	17218.31 [3125.0–86100.28]	**<0.001**	U	45,519
MII-3	81378.43 [12788.72–447878.2]	164297.4 [27366.1–794295.34]	**<0.001**	U	46,066
PNI	38.4 [32.71–44.24]	32.5 [27.15–40.05]	**<0.001**	U	73,565
PMI	2260.55 [1780.02–2897.45]	2060.0 [1463.7–2822.4]	**0.014**	U	56692.5
CAR	1.22 [0.28–3.82]	2.87 [0.98–6.58]	**<0.001**	U	41158.5
In ICU					
Dialyses n (%)	79 (16.5%)	88 (38.1%)	**<0.001**	Χ^2^	39.045
Sepsis n (%)	90 (18.8%)	74 (32.0%)	**<0.001**	Χ^2^	14.542
Inotrop n (%)	135 (28.2%)	176 (76.2%)	**<0.001**	Χ^2^	143.464
Vent n (%)	343 (71.8%)	227 (98.3%)	**<0.001**	Χ^2^	67.7748
AKI n (%)	76 (15.9%)	89 (38.5%)	**<0.001**	Χ^2^	43.2694

Continuous variables are presented as median [interquartile range] unless otherwise indicated. Age and HGB are presented as mean ± standard deviation. Bold values indicate statistical significance at *p* < 0.05. n: count; %: percentage; LOS, length of stay; DM, diabetes mellitus; HT, hypertension; CAD, coronary artery disease; COPD, chronic obstructive pulmonary disease; HF, heart failure; OnkoDis, oncologic disease; GCS, Glasgow Coma Scale; Crea, creatinine; eGFR, estimated glomerular filtration rate; AST, aspartate aminotransferase; ALT, alanine aminotransferase; WBC, white blood cell count; HGB, hemoglobin; PLT, platelet count; RDW, red cell distribution width; LYM, lymphocyte count; MONO, monocyte count; NEU, neutrophil count; PDW, platelet distribution width; MPV, mean platelet volume; PCT, plateletcrit; CRP, C-reactive protein; NLR, neutrophil-to-lymphocyte ratio; PLR, platelet-to-lymphocyte ratio; HALP, hemoglobin-albumin-lymphocyte-platelet score; SII, systemic immune-inflammation index; AISI, aggregate index of systemic inflammation; SIRI, systemic inflammation response index; MII, multi-inflammatory index; PNI, prognostic nutritional index; PMI, platelet mass index; CAR, C-reactive protein-to-albumin ratio; AKI, acute kidney injury.

Compared with survivors, non-survivors were significantly older (mean age 75.34 ± 13.25 vs. 69.77 ± 16.89 years; *p* < 0.001) and had lower Glasgow Coma Scale scores at emergency department admission (median 8.0 [IQR 3.0–13.0] vs. 14.0 [IQR 8.0–15.0]; *p* < 0.001). The prevalence of coronary artery disease (52.8% vs. 41.6%; *p* = 0.007) and oncologic disease (13.9% vs. 7.3%; *p* = 0.008) was higher among non-survivors. Sex distribution did not differ significantly between groups (*p* = 0.52).

### Laboratory parameters

Significant differences were observed between survivor and non-survivor groups for several laboratory parameters. Non-survivors had higher urea, creatinine, aspartate aminotransferase, alanine aminotransferase, red cell distribution width, and C-reactive protein levels, along with lower estimated glomerular filtration rate, hemoglobin, platelet count, and albumin levels (all *p* < 0.001; Table 2). Platelet indices, including mean platelet volume, platelet distribution width, and plateletcrit, also differed significantly between groups.

### Inflammatory and immuno-nutritional indices

Among inflammatory and nutritional indices, significant between-group differences were observed for neutrophil-to-lymphocyte ratio, hemoglobin-albumin-lymphocyte-platelet score, prognostic nutritional index, platelet mass index, CAR, and all MII variants (MII-1, MII-2, and MII-3; all *p* < 0.05; Table 2). No significant differences were observed for platelet-to-lymphocyte ratio, systemic immune-inflammation index, aggregate index of systemic inflammation, or systemic inflammation response index.

### Cause-specific Cox regression analyses

Age- and sex-adjusted SD-scaled cause-specific Cox proportional hazards analyses for in-hospital mortality are presented in Table 3. CAR was associated with in-hospital mortality (HR 1.268 per 1 SD increase; 95% CI 1.126–1.427; *p* < 0.001). MII-1 (HR 1.190; 95% CI 1.042–1.358; *p* = 0.010), MII-2 (HR 1.292; 95% CI 1.159–1.439; *p* < 0.001), and MII-3 (HR 1.212; 95% CI 1.087–1.353; *p* < 0.001) were also associated with mortality on a standardized scale. SIRI (HR 1.146; 95% CI 1.020–1.288; *p* = 0.022) and PNI (HR 0.641; 95% CI 0.546–0.752; *p* < 0.001) also showed significant associations. These results should be interpreted together with the ROC analyses, which demonstrated limited discriminatory performance for most indices.

**Table 3:** Age- and sex-adjusted cause-specific hazard ratios for in-hospital mortality after ICU admission (HR per 1 SD increase).

Index	Scale	Hazard Ratio	95% CI	*p*-value
NLR^‡^	per 1 SD (192.2)	1.138	0.996–1.300	0.057
PLR^‡^	per 1 SD (1286.3)	1.131	0.966–1.323	0.126
SII^‡^	per 1 SD (55381.6)	1.053	0.919–1.207	0.456
SIRI^‡^	per 1 SD (130.3)	**1.146**	1.020–1.288	0.022*
AISI^‡^	per 1 SD (23200.7)	1.090	0.971–1.223	0.143
MII-1^‡^	per 1 SD (2,299,189)	**1.190**	1.042–1.358	0.010*
MII-2^‡^	per 1 SD (15,544,455)	**1.292**	1.159–1.439	<0.001*
MII-3^‡^	per 1 SD (635,606,153)	**1.212**	1.087–1.353	<0.001*
CAR^‡^	per 1 SD (3.42)	**1.268**	1.126–1.427	<0.001*
HALP^‡^	per 1 SD (26.3)	1.011	0.868–1.178	0.888
PNI^‡^	per 1 SD (9.44)	**0.641**	0.546–0.752	<0.001*
PMI^‡^	per 1 SD (1070.9)	0.878	0.760–1.015	0.079

All models were adjusted for age and sex; age and sex coefficients are not shown because each index was evaluated in a separate adjusted model. Hazard ratios (HRs) are reported per 1 standard deviation (SD) increase to improve interpretability. All indices were winsorized at the 99th percentile prior to SD scaling. SD values are shown in parentheses in the Scale column. For indices with HR < 1, higher values are associated with lower mortality risk. Bold values and asterisks (*) indicate statistically significant results, defined as *p* < 0.05. ^‡^HR rescaled per 1 SD increase; original unit-based HR approaches 1.000 due to large numerical scale. NLR, neutrophil-to-lymphocyte ratio; PLR, platelet-to-lymphocyte ratio; SII, systemic immune-inflammation index; SIRI, systemic inflammation response index; AISI, aggregate index of systemic inflammation; MII-1/2/3, multi-inflammatory index variants; CAR, C-reactive protein-to-albumin ratio; HALP, hemoglobin-albumin-lymphocyte-platelet score; PNI, prognostic nutritional index; PMI, platelet mass index; CI, confidence interval; SD, standard deviation; ICU, intensive care unit.

In a sensitivity analysis including GCS score and oncological disease status as additional covariates, CAR remained associated with in-hospital mortality in an extended cause-specific Cox model (HR 1.060; 95% CI 1.025–1.096; *p* = 0.001). GCS score (HR 0.932; 95% CI 0.904–0.960; *p* < 0.001) and oncological disease status (HR 2.109; 95% CI 1.423–3.126; *p* < 0.001) were also associated with mortality; age and sex were not statistically significant in this expanded model.

### ROC curve analyses

**Figure 1:**
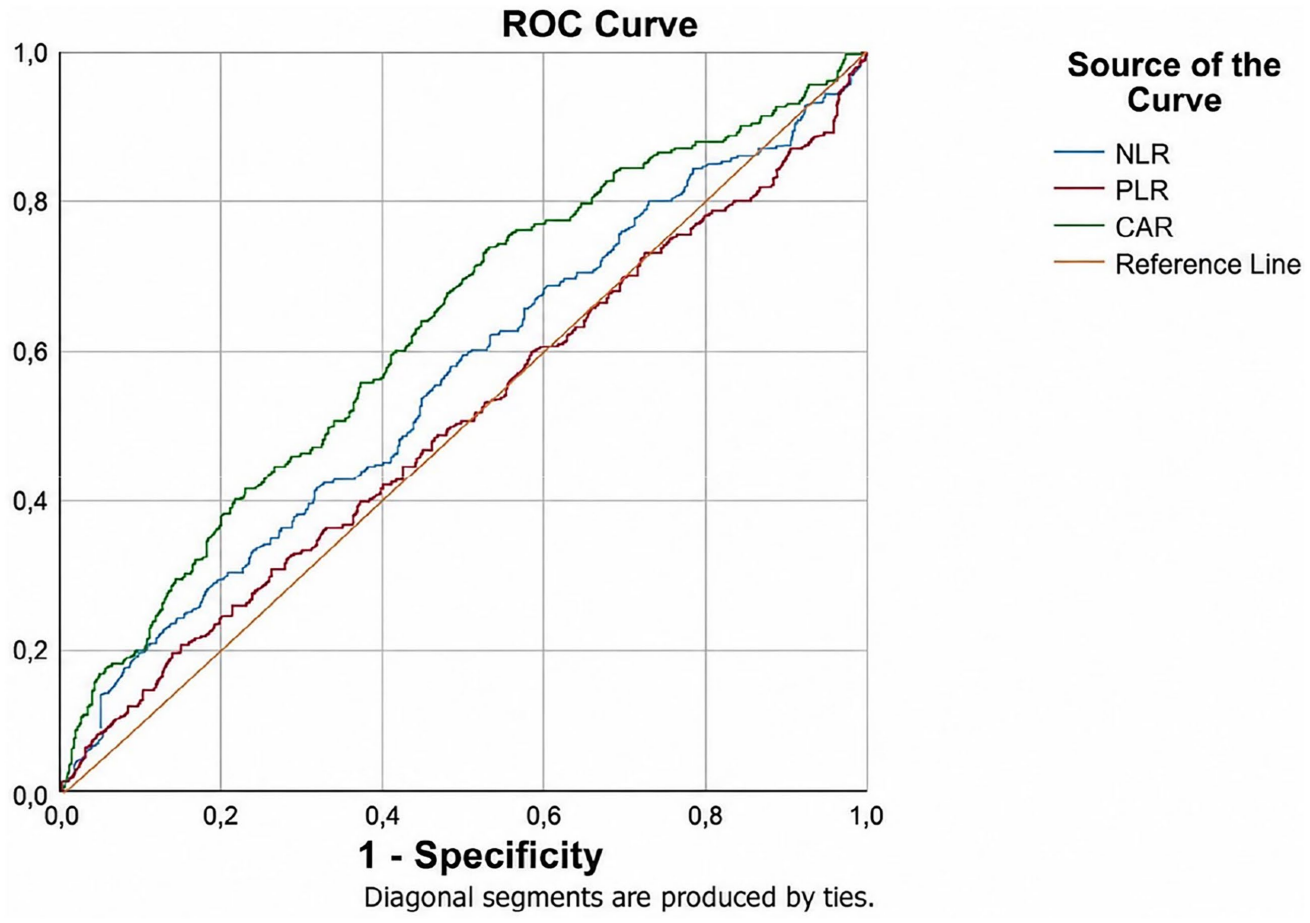
Receiver operating characteristic (ROC) curves of ratios in for in-hospital mortality. ROC curves for neutrophil-to-lymphocyte ratio (NLR, blue), platelet-to-lymphocyte ratio (PLR, red), and C-reactive protein-to-albumin ratio (CAR, green) in predicting in-hospital mortality. The diagonal line represents the reference line (AUC = 0.5). Greater deviation of the curve from the reference line indicates better discriminative ability.

**Figure 2:**
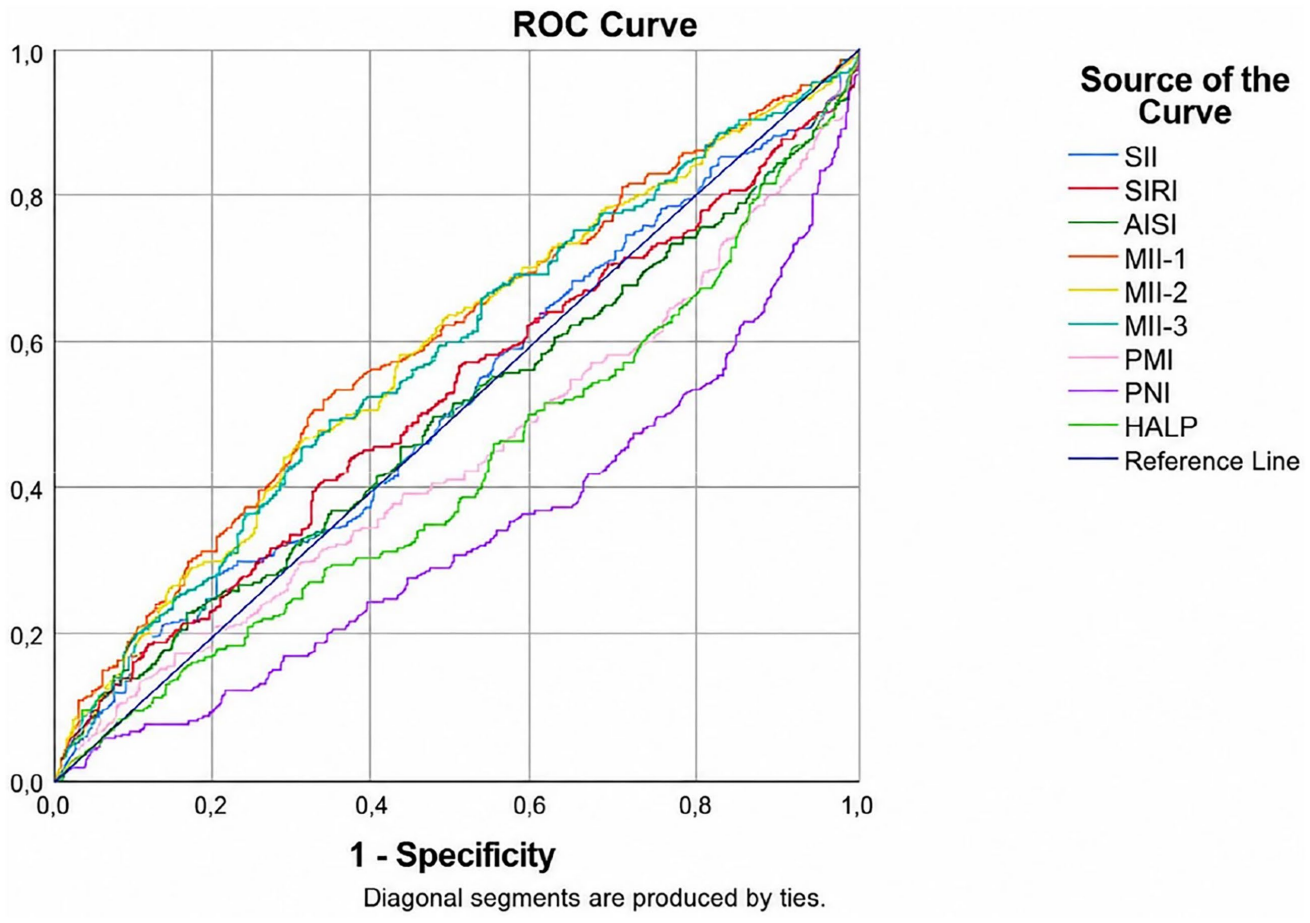
Receiver operating characteristic (ROC) curves of composite inflammatory and nutritional indices in predicting in-hospital mortality. ROC curves for systemic immune-inflammation index (SII, blue), systemic inflammation response index (SIRI, red), aggregate index of systemic inflammation (AISI, green), multi-inflammatory index 1 (MII-1, orange), multi-inflammatory index 2 (MII-2, yellow), multi-inflammatory index 3 (MII-3, cyan), platelet mass index (PMI, pink), prognostic nutritional index (PNI, purple), and hemoglobin-albumin-lymphocyte-platelet score (HALP, light green) in predicting in-hospital mortality. Lower values of PMI, PNI, and HALP were observed or treated as inverse-direction markers in the ROC analysis; therefore, corrected AUC values for these indices are reported in Table 4.

Receiver operating characteristic curve analyses evaluating the discriminative performance of inflammatory and nutritional indices for in-hospital mortality are shown in Table 4 and Figures 1, 2. The area under the curve, optimal cut-off values, sensitivity, specificity, and corresponding 95% confidence intervals are reported for each index.

**Table 4:** Receiver operating characteristic (ROC) analysis results for the discriminative performance of ratios and indices for in-hospital mortality,

Index	Cut-off	Sensitivity	Specificity	AUC	95% CI	*p*-value
NLR	20.6179	0.294	0.805	0.558	0.512 to 0.604	**0.012**
PLR	449.0883	0.208	0.849	0.505	0.458 to 0.552	0.820
CAR	1.0765	0.740	0.469	0.627	0.583 to 0.671	**<0.001**
SII	4544.29	0.286	0.795	0.517	0.469 to 0.564	0.486
SIRI	21.80	0.166	0.900	0.518	0.470 to 0.567	0.444
AISI	7605.81	0.124	0.936	0.493	0.445 to 0.542	0.777
MII-1	468.23	0.622	0.515	0.595	0.549 to 0.642	**<0.001**
MII-2	13612.70	0.571	0.575	0.584	0.537 to 0.631	**<0.001**
MII-3	147179.74	0.525	0.611	0.579	0.532 to 0.626	**0.001**
PMI	1702.35	0.782	0.346	0.558	0.510 to 0.607	**0.014**
PNI	31.45	0.825	0.447	0.663	0.618 to 0.709	**<0.001**
HALP	11.214	0.442	0.692	0.567	0.529 to 0.624	**0.004**

Cut-off values were derived from receiver operating characteristic (ROC) curve analysis. Sensitivity and specificity are presented for the optimal cut-off of each marker for in-hospital mortality. The area under the curve (AUC) is shown with its 95% confidence interval, and p-values indicate the statistical significance of each AUC compared with the null hypothesis (AUC = 0.5). For PMI, PNI, and HALP, lower values were associated with mortality; therefore, the ROC direction was reversed so that AUC values above 0.5 consistently indicate discrimination for mortality. Bold values indicate statistically significant results, defined as *p* < 0.05. NLR, neutrophil-to-lymphocyte ratio; PLR, platelet-to-lymphocyte ratio; HALP, hemoglobin-albumin-lymphocyte-platelet score; SIRI, systemic inflammation response index; AISI, aggregate index of systemic inflammation; SII, systemic immune-inflammation index; MII-1/2/3, multi-inflammatory index (1, 2, and 3); PNI, prognostic nutritional index; PMI, platelet mass index; CAR, C-reactive protein-to-albumin ratio.

## Discussion

This study evaluated the association between admission-based inflammatory and immuno-nutritional indices and in-hospital mortality in critically ill patients admitted from the emergency department to the intensive care unit. CAR showed the most consistent association with mortality in age and sex-adjusted and sensitivity analyses. MII variants were also associated with mortality after SD-scaling, indicating that the previous unit-based hazard ratios were difficult to interpret because of their large numerical ranges. However, the ROC-derived AUC values of CAR and MII variants remained modest or low. Therefore, these indices should be interpreted as adjunctive markers of inflammatory burden rather than standalone prognostic tools.

After adjustment for age and sex and accounting for discharge as a competing event, SD-scaled cause-specific Cox models showed that CAR was associated with in-hospital mortality (HR 1.268 per 1 SD increase; 95% CI 1.126–1.427; *p* < 0.001). This relationship was consistent across sensitivity analyses, including GCS score and oncologic disease status. Albumin was excluded from this model to avoid collinearity, as it is a direct component of the CAR formula. Biologically, CAR may reflect the combined effect of acute inflammation and reduced physiological or nutritional reserves, since CRP increases during systemic inflammation, while albumin decreases during severe illness due to negative acute phase response, impaired synthesis, and capillary leakage (11, 13, 16).

Our findings regarding CAR are largely consistent with previous intensive care studies summarized in Supplementary Table 1. The AUC (0.627) observed in the current cohort is comparable to those reported by Ranzani et al. (0.612), Park et al. (0.594), and Żerdziński et al., who also reported modest discriminative performance in ICU populations (14–16). These findings support the interpretation of CAR as a mortality-associated, admission-based marker, while also confirming its limited discriminatory performance at the individual level.

In this study, PNI and HALP values were higher among survivors than non-survivors in unadjusted analyses, suggesting a possible association between impaired nutritional status, reduced immune reserve, and adverse outcomes in critically ill patients. PNI and HALP have been investigated in oncologic, surgical, septic, and cardiovascular populations (22–26). PNI showed the highest AUC value (0.663) among the assessed indices, numerically exceeding CAR (0.627), and was also associated with mortality in SD-scaled Cox analyses. However, PNI and CAR likely reflect different biological dimensions: PNI primarily represents nutritional reserve and immune competence, whereas CAR reflects acute inflammatory burden related to physiological reserve. Since the discriminative performance of PNI remained modest and the analyses were exploratory in nature, this finding should be interpreted as hypothesis-generating rather than evidence of standalone prognostic utility. The lack of standardized threshold values and the use of measurements taken only upon hospital admission further limit the immediate clinical applicability of nutritional indices in routine risk stratification (27).

Other CBC-derived inflammatory indices, including NLR, PLR, SII, SIRI, and AISI, demonstrated limited discriminative performance in this heterogeneous emergency department-ICU cohort. While some of these indices were statistically associated with mortality in SD-scaled Cox analyses, AUC values derived from ROC remained low or unacceptable. These findings differ from some reports in selected disease-specific populations where indices such as NLR have shown stronger performance, but are consistent with studies suggesting limited prognostic value of CBC-derived indices in general ICU populations (3, 28–34). This variability may reflect differences in patient selection, outcome definitions, timing of biomarker measurement, and model adjustment strategies. Therefore, these indices should be interpreted as contextual, admission-based markers of inflammatory burden, rather than definitive tools for individual-level risk discrimination.

ROC analyses showed limited discriminatory performance across the evaluated indices, as reflected by generally low AUC values. Optimal cutoff values derived from ROC analyses are presented in Table 4 as exploratory reference points for future comparative research: CAR ≥1.077 (sensitivity 74.0%, specificity 46.9%, AUC 0.627), MII-1 ≥ 468.23 (sensitivity 62.2%, specificity 51.5%, AUC 0.595), MII-2 ≥ 13,612.70 (sensitivity 57.1%, specificity 57.5%, AUC 0.584), and MII-3 ≥ 147,179.74 (sensitivity 52.5%, specificity 61.1%, AUC 0.579). Although SD-scaled Cox analyses improved the interpretability of hazard ratios and demonstrated statistically significant associations for MII variants, these associations did not translate into clinically meaningful individual-level discrimination. Therefore, these threshold values should be considered descriptive and hypothesis-generating rather than clinically validated and external prospective validation is required before clinical use.

Overall, the findings support a multidimensional interpretation of inflammatory and immuno-nutritional indices in critically ill patients. Current composite biomarkers, including PNI, HALP, PIV, Naples score, and other complete blood count-based indices, have been evaluated in sepsis, cardiovascular disease, and other acute care populations and may reflect different aspects of host response, such as inflammation, immune cell redistribution, nutritional reserve, and physiological stress (12, 32, 35).

The use of a single admission measurement is both a design feature and a limitation of this study. This approach reflects emergency department practice, where early risk assessment is often required before longitudinal biomarker data are available. However, in patients with prolonged intensive care or hospital stays, admission values may not capture subsequent inflammatory dynamics, treatment response, or complications. Therefore, the findings should be interpreted as reflecting admission-based risk information rather than time-dependent prognostic performance.

Subgroup analyses by major diagnostic categories were not performed because of the limited number of patients in individual diagnostic groups and the resulting insufficient statistical power. The present cohort was obtained from a general intensive care unit that admitted heterogeneous patients from the emergency department, including those with respiratory failure, sepsis or infection-related conditions, renal-metabolic disorders, and a decreased level of consciousness. Since patients with primary cardiac or neurological indications are generally managed in their respective specialized intensive care units at our institution, these patient groups may be underrepresented in the present cohort. Future studies with larger, diagnosis-specific cohorts are needed to evaluate whether the discriminatory performance of CAR and other inflammatory indices differs among clinical subgroups.

## Limitations

This study has several important limitations that should be acknowledged. First, although inflammatory biomarkers are widely used in critical care, existing evidence suggests that no single inflammatory marker is sufficient to accurately characterize mortality risk in critically ill patients. Consequently, combinations of biomarkers and clinical parameters may be required to improve prognostic performance.

Second, the present study did not include a comparison with established prognostic scoring systems such as APACHE II and SOFA scores. Although APACHE II and SOFA are widely validated for mortality prediction in critically ill patients, they require multiple physiological and laboratory parameters obtained within the first 24 h of ICU admission. The present study specifically focused on inflammatory and immuno-nutritional indices derived from laboratory parameters available at emergency department admission in order to evaluate their early prognostic utility. Furthermore, due to the retrospective design, several variables necessary for reliable calculation of APACHE II and SOFA scores were inconsistently documented in the electronic medical records. Restricting the analysis only to patients with complete APACHE II data could have reduced the sample size and introduced selection bias. Future prospective studies incorporating APACHE II and SOFA may provide more comprehensive prognostic comparisons.

Third, the indices were calculated from a single admission time point. In critical illness, inflammatory and nutritional parameters are dynamic; without serial measurements or trajectory analyses, it is not possible to determine whether these indices capture stable prognostic information versus transient physiologic variation.

Fourth, since patients with primary cardiac or neurological indications are generally admitted to the relevant specialized intensive care units at our institution, these patients may be underrepresented in the current general intensive care cohort.

While SD-scaled Cox analyses improve the interpretability of hazard ratios for indices with wide numerical ranges, these analyses do not eliminate the limited discriminatory performance observed in ROC analyses. Therefore, statistically significant associations after rescaling should not be interpreted as evidence of clinical benefit.

Finally, the potential influence of comorbid conditions on inflammatory responses could not be fully accounted for, and the clinical utility of MII-based indices across different patient populations requires further validation.

### Future perspective

The findings of this study highlight the growing interest in integrating composite biomarkers into early risk assessment strategies in emergency and critical care settings. Multi-parameter indices such as MII may offer a quantitative representation of systemic inflammatory burden, but their potential role as adjunctive tools requires prospective validation against established severity scores.

However, given that most available evidence, including the present study, is derived from retrospective and single-center cohorts, further prospective, multicenter studies involving diverse intensive care populations are needed. Such studies should focus on validating the prognostic value of these indices, defining standardized cut-off values, and evaluating their incremental contribution beyond established severity scores.

Future research may also explore the integration of composite inflammatory indices into structured clinical decision-support frameworks, provided that their clinical utility and reliability are demonstrated through rigorous prospective validation. Only through such validation can these indices be considered for broader clinical implementation.

## Data Availability

The datasets generated and/or analyzed during the current study are not publicly available due to institutional data protection policies but are available from the corresponding author upon reasonable request.
