# Supplementary material for: Admission inflammatory and immuno-nutritional indices and in-hospital mortality in emergency department patients admitted to intensive care: a retrospective cohort study
Source: Front Med (Lausanne). 2026 Jul 20;13:1903440. doi: 10.3389/fmed.2026.1903440 (PMC13429788; doi:10.3389/fmed.2026.1903440)
Supplement: Supplementary file 1 [file Supplementary_file_1.docx]

Supplementary Table 1. Comparison of reported CAR performance across critical care studies

| **Study** | **Population (Year)** | **Outcome** | **N** | **CAR cut-off** | **AUC** | **Senst.** | **Spec.** |
| --- | --- | --- | --- | --- | --- | --- | --- |
| Ranzani et al. [14] | Patients with sepsis (2013) | 90-day mortality | 334 | 8.7 | 0.612 | 54.0% | 66.0% |
| Park et al. [15] | Critically ill ICU patients (2018) | 28-day mortality | 875 | 34.3 | 0.594 | 64.2% | 52.7% |
| Żerdziński et al. [13] | Tertiary cardiac ICU cohort (2026) | ICU Mortality | 137 | 3.176 | 0.677 | 68.7% | 65.7% |
| Present study | Heterogeneous ED-to-ICU population | In-hospital mortality | 709 | 1.077 | 0.627 | 74.0% | 46.9% |

CAR performance differed across studies because of variation in patient population, timing of CAR measurement, outcome definition, follow-up duration, ICU type, and laboratory unit conventions. Therefore, CAR cut-off values and AUCs should be compared cautiously. In the present study, CAR was calculated from laboratory values obtained at emergency department admission and was evaluated for in-hospital mortality. Abbreviations: AUC, area under the receiver operating characteristic curve; CAR, C-reactive protein-to-albumin ratio; ED, emergency department; ICU, intensive care unit; N, number of patients or admissions; Sens., sensitivity; Spec., specificity.

| **Variable** | **HR** | **95% CI** | **p-value** |
| --- | --- | --- | --- |
| Age | 1.006 | 0.996–1.016 | 0.239 |
| Sex | 0.824 | 0.631–1.076 | 0.155 |
| **CAR** | 1.060 | 1.025–1.096 | **0.001^*^** |
| **GCS** | 0.932 | 0.904–0.960 | **<0.001^*^** |
| **Oncologic disease** | 2.109 | 1.423–3.126 | **<0.001^*^** |

Supplementary Table 2. Sensitivity analysis using an extended cause-specific Cox model for in-hospital mortality

The extended cause-specific Cox model included age, sex, CAR, GCS score, and oncologic disease status. Albumin was excluded to avoid collinearity because it is a direct component of the CAR formula. HR values are reported per one-unit increase for continuous variables and relative to the reference category for categorical variables. Abbreviations: CAR, C-reactive protein-to-albumin ratio; CI, confidence interval; GCS, Glasgow Coma Scale; HR, hazard ratio.

Table 3. Age- and sex-adjusted cause-specific hazard ratios for in-hospital mortality after ICU admission (HR per 1 SD increase)

| Index | Scale | Hazard Ratio | 95% CI | p-Value |
| --- | --- | --- | --- | --- |
| NLR^‡^ | per 1 SD (192.2) | 1.138 | 0.996–1.300 | 0.057 |
| PLR^‡^ | per 1 SD (1286.3) | 1.131 | 0.966–1.323 | 0.126 |
| SII^‡^ | per 1 SD (55381.6) | 1.053 | 0.919–1.207 | 0.456 |
| **SIRI**^‡^ | per 1 SD (130.3) | **1.146** | 1.020–1.288 | **0.022*** |
| AISI^‡^ | per 1 SD (23200.7) | 1.090 | 0.971–1.223 | 0.143 |
| **MII-1**^‡^ | per 1 SD (2,299,189) | **1.190** | 1.042–1.358 | **0.010*** |
| **MII-2**^‡^ | per 1 SD (15,544,455) | **1.292** | 1.159–1.439 | **<0.001*** |
| **MII-3**^‡^ | per 1 SD (635,606,153) | **1.212** | 1.087–1.353 | **<0.001*** |
| **CAR**^‡^ | per 1 SD (3.42) | **1.268** | 1.126–1.427 | **<0.001*** |
| HALP^‡^ | per 1 SD (26.3) | 1.011 | 0.868–1.178 | 0.888 |
| **PNI**^‡^ | per 1 SD (94.4) | **0.641** | 0.546–0.752 | **<0.001*** |
| PMI^‡^ | per 1 SD (1070.9) | 0.878 | 0.760–1.015 | 0.079 |

All models were adjusted for age and sex. All indices were winsorized at the 99th percentile prior to SD scaling. Hazard ratios (HRs) are reported per 1 standard deviation (SD) increase for indices with large numerical ranges to improve interpretability. SD values are shown in parentheses in the Scale column. For protective indices (PNI, HALP, PMI), HR < 1 indicates that higher values are associated with lower mortality risk. * p < 0.05 considered statistically significant. ‡ HR rescaled per 1 SD increase; original unit-based HR approaches 1.000 due to large numerical scale. * CAR also remained independently associated with in-hospital mortality in a sensitivity analysis additionally adjusted for GCS score and oncologic disease status (HR 1.060; 95% CI 1.025–1.096; p = 0.001). Abbreviations: NLR, neutrophil-to-lymphocyte ratio; PLR, platelet-to-lymphocyte ratio; SII, systemic immune-inflammation index; SIRI, systemic inflammation response index; AISI, aggregate index of systemic inflammation; MII-1/2/3, multi-inflammatory index variants; CAR, C-reactive protein-to-albumin ratio; HALP, hemoglobin-albumin-lymphocyte-platelet score; PNI, prognostic nutritional index; PMI, platelet mass index; CI, confidence interval; SD, standard deviation; ICU, intensive care unit.

Supplementary Table 4. Extended sensitivity analysis of cause-specific hazard ratios for in-hospital mortality after ICU admission, adjusted for age, sex, GCS score, and oncologic disease status

| Index / Covariate | Scale | HR | 95% CI | p-Value |
| --- | --- | --- | --- | --- |
| ***Fixed Covariates,consistent across all models*** | | | | |
| Age | per 1 year | 1.006 | 0.996–1.016 | 0.239 |
| Sex (Male vs Female) | — | 0.824 | 0.631–1.076 | 0.155 |
| **GCS** | per 1 point | **0.932** | 0.904–0.960 | **<0.001*** |
| **Oncologic disease** | yes vs no | **2.109** | 1.423–3.126 | **<0.001*** |
| ***Inflammatory and Immuno-nutritional Indices (each entered separately per SD)*** | | | | |
| NLR | per 1 SD (192.2) | 1.132 | 0.990–1.294 | 0.071 |
| PLR | per 1 SD (1,286.3) | 1.167 | 0.999–1.363 | 0.051 |
| SII | per 1 SD (55,381.6) | 1.083 | 0.952–1.233 | 0.224 |
| **SIRI** | per 1 SD (130.3) | **1.178** | 1.062–1.307 | **0.002*** |
| **AISI** | per 1 SD (23,200.7) | **1.134** | 1.025–1.254 | **0.015*** |
| **MII-1** | per 1 SD (2,299,189) | **1.185** | 1.026–1.369 | **0.021*** |
| **MII-2** | per 1 SD (15,544,455) | **1.306** | 1.176–1.450 | **<0.001*** |
| **MII-3** | per 1 SD (635,606,153) | **1.233** | 1.111–1.369 | **<0.001*** |
| **CAR** | per 1 SD (3.42) | **1.220** | 1.081–1.378 | **0.001*** |
| HALP | per 1 SD (26.3) | 0.974 | 0.844–1.124 | 0.715 |
| **PNI** | per 1 SD (94.4) | **0.686** | 0.586–0.803 | **<0.001*** |
| PMI | per 1 SD (1,070.9) | 0.890 | 0.766–1.034 | 0.127 |

Each inflammatory or immuno-nutritional index was entered separately into individual models alongside the fixed covariates (age, sex, GCS score, oncologic disease). Hazard ratios (HRs) are reported per 1 standard deviation (SD) increase; SD values are shown in parentheses in the Scale column. Winsorization at the 99th percentile was applied to indices with extreme outliers prior to SD scaling. Green shading indicates statistically significant associations (p < 0.05). For protective indices (PNI, HALP, PMI), HR < 1 indicates that higher values are associated with lower mortality risk. * p < 0.05 considered statistically significant. Abbreviations: HR, hazard ratio; CI, confidence interval; SD, standard deviation; GCS, Glasgow Coma Scale; NLR, neutrophil-to-lymphocyte ratio; PLR, platelet-to-lymphocyte ratio; SII, systemic immune-inflammation index; SIRI, systemic inflammation response index; AISI, aggregate index of systemic inflammation; MII-1/2/3, multi-inflammatory index variants 1, 2, and 3; CAR, C-reactive protein-to-albumin ratio; HALP, hemoglobin-albumin-lymphocyte-platelet score; PNI, prognostic nutritional index; PMI, platelet mass index.
